# Novel Immune Checkpoint Inhibitor and Antibody–Drug Conjugate Approaches in the Perioperative Management of Muscle-Invasive Bladder Cancer

**DOI:** 10.3390/curroncol33030162

**Published:** 2026-03-12

**Authors:** Joseph Vento, Tian Zhang, Yair Lotan, Solomon Woldu, Qian Qin

**Affiliations:** 1 Department of Internal Medicine, Division of Hematology & Oncology, University of Texas Southwestern Medical Center, Dallas, TX 75390, USA; 2 Department of Urology, University of Texas Southwestern Medical Center, Dallas, TX 75390, USA

**Keywords:** muscle-invasive bladder cancer, urothelial cancer, immunotherapy, immune checkpoint inhibitors, antibody–drug conjugates, perioperative systemic therapy

## Abstract

Immune checkpoint inhibitors and antibody drug conjugate combinations have revolutionized the management of patients with advanced and metastatic urothelial carcinoma, offering unprecedented survival outcomes. These treatments are now moving into earlier stages of disease, including perioperative treatments for patients with muscle-invasive bladder cancer planning for curative-intent radical cystectomy. In this setting, there are now standard-of-care options for adjuvant immune checkpoint inhibitors with or without prior neoadjuvant chemotherapy, perioperative immune checkpoint plus cytotoxic chemotherapy combinations, and perioperative immune checkpoint inhibitor plus antibody drug conjugate combinations. This review will evaluate key clinical trials that led to modern standards of care involving these classes of drugs and highlight ongoing clinical trials that may further shift treatment paradigms for muscle-invasive bladder cancer. Key efficacy and toxicity considerations will be reviewed, and available evidence for biomarkers will be evaluated. As immune checkpoint inhibitors and antibody drug conjugates continue to demonstrate improved outcomes across the spectrum of bladder cancer treatment, understanding their role in the muscle-invasive disease state is crucial to managing patients with this condition.

## Simple Summary

The approach to treating muscle-invasive bladder cancer is shifting toward immunotherapy-based regimens and increasing use of antibody drug conjugates before and after surgery. In this paper, we review the key studies that led to these shifts in the perioperative management of muscle-invasive bladder cancer.

## 1. Introduction

In 2025, nearly 85,000 new cases of bladder cancer were diagnosed in the United States, with 35% of cases presenting at a localized stage and over 90% having urothelial histology [1,2]. When bladder cancer invades into or beyond the muscularis propria layer of the bladder wall, it is referred to as muscle-invasive bladder cancer (MIBC). Treatment approaches for patients with MIBC must take into account the high risk of local recurrence and potential for metastatic spread [3]. Furthermore, there is a significant risk of understaging of patients with MIBC, with up to 40% of patients getting understaged [4]. Patients with unrecognized non-organ-confined disease have a lower chance of cure with local treatment alone.

Prior to 2020, the standard of care for the management of clinically localized, urothelial MIBC included two main options: neoadjuvant cisplatin-based chemotherapy followed by radical cystectomy, or trimodal therapy combining maximal resection, radiation therapy, and radiosensitizing chemotherapy [5]. While neoadjuvant cisplatin-based chemotherapy is recommended in the guidelines, less than 50% of patients undergoing cystectomy receive neoadjuvant chemotherapy for various reasons, including patient and physician preference, renal function and other comorbidities, and a small perceived survival benefit [6]. A significant advancement in this space emerged with the adoption of immune checkpoint inhibitor (ICI) immunotherapies into MIBC clinical trials. In patients eligible for and electing to undergo radical cystectomy, there is evidence for ICI therapy administered either in a sandwich approach (together with chemotherapy in the neoadjuvant setting and continuing in the adjuvant setting following surgery) or in the adjuvant setting with or without prior neoadjuvant chemotherapy [7]. For patients with localized bladder cancer who qualify and elect for trimodal therapy, the results of ongoing randomized trials such as SWOG/RTG 1806 (chemoradiotherapy with or without the PD-L1-targeting ICI atezolizumab in MIBC) and KEYNOTE-922 (chemoradiotherapy with or without the PD-1-targeting ICI pembrolizumab in MIBC) may further elucidate the role of ICI [8,9]. For this review, we focus on perioperative treatment of bladder cancer in patients with MIBC electing for radical cystectomy and summarize the trials that have led to the adoption of ICI strategies in this space.

Another advancement in bladder cancer systemic therapies came with the development of antibody–drug conjugates (ADCs) and ADC combinations, which demonstrated markedly improved clinical outcomes in advanced bladder cancer relative to cisplatin-based chemotherapy. Most notably in the EV-302 trial, the ADC, enfortumab vedotin (EV), in combination with the ICI, pembrolizumab, led to drastic improvement in median overall survival (OS) relative to platinum-based chemotherapy (31.5 months vs. 16.1 months, hazard ratio [HR] 0.47, 95% confidence interval [CI] 0.38–0.58, *p* < 0.001) [10]. More recently, in the EV-303/KEYNOTE-905 and EV-304/KEYNOTE-B15 trials, perioperative EV/pembrolizumab in patients with MIBC demonstrated improved event-free survival (EFS), OS, and pathologic complete response (pCR) when compared to radical cystectomy with or without platinum-based chemotherapy [11,12,13]. Early phase and ongoing trials also indicate the promise of tumor-associated calcium signal transducer 2 (Trop-2) and epidermal growth factor receptor 2 (HER2)-targeting ADCs [14,15,16]. Given the promising activity of perioperative ADCs in MIBC patients, we review here the reported and ongoing ADC perioperative trials that continue to expand systemic therapy options for MIBC patients.

## 2. Results

A summary of completed and ongoing MIBC clinical trials, including perioperative ICIs with or without chemotherapy plus radical cystectomy, is shown in Table 1. A summary of completed and ongoing MIBC clinical trials, including perioperative ADCs alone or in combination with ICIs plus radical cystectomy, is shown in Table 2. Potential limitations and biases of relevant phase III trials in this space are summarized in Table 3.

### 2.1. ICIs for Perioperative Management of MIBC

The first published clinical trials investigating perioperative ICIs in MIBC involved phase II studies administering ICIs in the neoadjuvant setting, with pathologic complete response (pCR) as the primary outcome. The largest representative studies are the PURE-01 and ABACUS studies, which demonstrated pCR rates of 36.8% and 31% with neoadjuvant pembrolizumab and atezolizumab, respectively, with few patients having surgery delayed due to complications of therapy [26,27]. Several phase II studies investigating neoadjuvant combination ICI therapies, including the NABUCCO trial with ipilimumab/nivolumab (CTLA-4 and PD-1-targeting ICIs, respectively) and the NCT02812420 trial with durvalumab/tremelimumab (PD-L1 and CTLA-4-targeting ICIs, respectively), demonstrated higher pCR rates (46% in NABUCCO cohort 1 and 37.5% in NCT02812420), though with higher rates of immune-related complications (55% and 21% grade 3+ toxicities, respectively) [39,40].

The next wave of reported ICI trials included randomized phase III trials investigating adjuvant ICI with or without prior neoadjuvant chemotherapy. The first of these published trials, IMvigor010, investigated adjuvant atezolizumab for one year vs. placebo and was negative for its primary outcome of disease-free survival (19.4 vs. 16.6 mo, HR 0.89, 95% CI 0.74–1.08, *p* = 0.24). However, subsequent post hoc analysis identified circulating tumor DNA (ctDNA) as a biomarker for improved DFS with atezolizumab (HR 0.58, 95% CI 0.43–0.79 in the ctDNA-positive subgroup) [41]. Subsequently, the phase III IMvigor011 study prospectively investigated adjuvant atezolizumab vs. placebo only in patients with ctDNA-positive disease after surgery (based on a tumor-informed ctDNA platform) and reported a significant improvement in DFS relative to placebo (9.9 vs. 4.8 mo, HR 0.64, 95% CI 0.47–0.87; *p* = 0.0047) [20].

For an alternative ICI, the CheckMate 274 trial investigated adjuvant nivolumab for one year vs. placebo and was positive for its primary outcome of DFS, with long-term outcomes demonstrating a median DFS of 21.9 vs. 11.0 months with a HR of 0.74 (95% CI 0.61–0.90). This trial also initially demonstrated improved OS, though the most recent update reported a mOS of 75.0 vs. 50.1 months and a HR of 0.83 (95% CI 0.67–1.02) [18]. Of interest, recent exploratory analysis in this trial highlights tumor cell PD-L1 expression, CD4 gene expression, 25-IFNγ gene expression signatures, and tumor mutational burden (TMB) as potential biomarkers for DFS benefit from nivolumab. Finally, the AMBASSADOR trial investigated adjuvant pembrolizumab versus placebo and was positive for the primary DFS outcome (mDFS of 29.6 vs. 14.2 mo, HR 0.73 (95% CI 0.59–0.90, *p* = 0.003)) [19]. Though the OS data for AMBASSOR remains immature, reported data shows a non-significant HR of 0.98 (95% CI 0.76–1.26). In these studies, 43–65% of patients received neoadjuvant cisplatin-based chemotherapy. The inclusion criteria for cancer stage were similar across adjuvant ICI trials, including T2 disease, only if a patient had received neoadjuvant chemotherapy (ypT2), and all trials allowed pathologically confirmed, regional node-positive disease (pN1). Grade 3 and higher adverse events, mainly immune-mediated, were consistently higher in the intervention arms of all phase III adjuvant ICI trials than in the placebo arms. Ongoing trials such as the phase II/III MODERN trial are investigating further on ctDNA stratification in the adjuvant ICI setting, where patients with postop ctDNA-negative disease are randomized to nivolumab versus ctDNA surveillance, and patients with postop ctDNA-positive disease are randomized to nivolumab with or without relatlimab (LAG3-targeting ICI therapy) [29].

In the meantime, ICIs have also moved into the neoadjuvant space, most frequently using the “sandwich” perioperative approach where ICI is given both prior to (with chemotherapy) and post-surgery. Several phase II trials investigated this approach, including the AURA Oncodistinct-004 trial [24], combining neoadjuvant avelumab with chemotherapy in both cisplatin-eligible and cisplatin-ineligible patients, the SAKK 06/17 trial [25] combining sandwich durvalumab combination strategies with cisplatin-based chemotherapy, the LCCC1520 study [42] combining sandwich pembrolizumab with cisplatin-based chemotherapy, and the HCRN GU14-188 trial [43] combining sandwich pembrolizumab/chemotherapy in both cisplatin-eligible and cisplatin-ineligible populations. The pCR rates in these trials varied from 32 to 58%.

Following these phase II trials, NIAGARA was the first large, phase III trial to report on perioperative chemoimmunotherapy. The trial investigated four cycles of neoadjuvant gemcitabine/cisplatin with or without perioperative durvalumab (neoadjuvant: four cycles; adjuvant: eight cycles), with dual primary outcomes of EFS and OS [17]. Importantly, split-dose cisplatin was allowed for patients with a glomerular filtration rate (GFR) of 40–60 mL/min/1.73 m^2^. Median EFS was not reached in the durvalumab combination population vs. 46.1 months in the chemotherapy arm, with a HR of 0.68 (95% CI 0.56–0.82, *p* < 0.001). Median OS was not reached in either arm but showed a hazard ratio of 0.75 (95% CI 0.59–0.93, *p* = 0.01) favoring the chemoimmunotherapy arm. EFS rates at 24 months were 67.8% vs. 59.8%, and OS rates at 24 months were 82.2% vs. 75.2%. Grade 3 or higher toxicities were 69.4% in the intervention arm (with 20.9% immune-related adverse events) vs. 67.5% in the chemotherapy alone arm. Delay in surgery related to adverse events was similar between the two arms (1.7% vs. 1.1%). Health-related quality of life analysis did not demonstrate detriment with the addition of durvalumab in this trial [44]. This compelling data led to the FDA approval of durvalumab–chemotherapy combination in March 2025. A post hoc tumor-informed ctDNA analysis of NIAGARA highlighted the prognostic value of this biomarker at various clinical timepoints, including patients having ctDNA clearance from baseline to pre-cystectomy (after either arm of neoadjuvant systemic therapy), who had improved EFS outcomes relative to patients without ctDNA clearance [45]. The ongoing KEYNOTE-866 trial is investigating the combination of perioperative pembrolizumab with chemotherapy in a similar population [28].

Other novel perioperative immunotherapy combination strategies are important to highlight, including ICI and local intravesical therapies or cancer vaccines. With the expansion of intravesicular localized therapy options in the non-muscle-invasive bladder cancer (NMIBC) space, the phase II SunRISe-4 trial investigated cetrelimab (PD-1 targeting ICI) with or without TAR-200 (an intravesical gemcitabine delivery system) in MIBC. The trial demonstrated improved primary endpoint of pCR (42% vs. 23%) and key exploratory endpoint of pathologic overall response rate (60% vs. 35%) in the TAR-200/cetrelimab cohort when compared to the cetrelimab cohort. Grade 3 or higher treatment-related adverse events were reported in 11% of the TAR-200 plus cetrelimab arm and 5% of the cetrelimab arm, with the most common event being hematuria in 3% (two patients) in the intervention arm [22]. The ongoing SAKK 06/19 (NCT04630730) is investigating a novel recombinant intravesical Bacillus Calmette–Guérin (BCG) vaccine given in 3 doses with perioperative chemoimmunotherapy (neoadjuvant gemcitabine–cisplatin with sandwich perioperative atezolizumab), with early efficacy data demonstrating a pCR rate of at least 9/21 (42.9%) with 9% grade 3+ toxicity related to BCG [46]. Furthermore, the ongoing Interpath-005 trial cohort is examining adjuvant pembrolizumab with or without a personalized mRNA vaccine (intismeran autogene or V940) delivered in a similar sandwich perioperative approach [30].

### 2.2. ADCs and ADC Combination Therapies for Perioperative Management of MIBC

Several ADCs have been evaluated in the treatment of urothelial carcinoma, including those directed against Nectin-4, TROP2, and HER2. EV is a human monoclonal antibody Nectin-4-targeting ADC, with a protease-cleavable linker and using monomethyl auristatin E (MMAE), a microtubule-disrupting agent, as its payload. Sacituzumab govitecan (SG) is a human monoclonal antibody TROP2-targeting ADC, with a hydrolysable linker to a SN-38 (metabolite of irinotecan) payload. Both targets are expressed in the majority of urothelial cancers, and studies to date have demonstrated some correlations of NECTIN4 gene amplification and Nectin-4 expression levels by immunohistochemistry (IHC) to tumor response to EV, but have not found such a correlation for TROP2 expression for SG [47,48]. Of note, membranous Nectin-4 expression levels have been shown to decrease in metastatic UC relative to primary tumors, highlighting a potential advantage of using this therapy in earlier-line settings. HER2 expression in MIBC can vary by selection of testing methodology and cutoff threshold for positive testing, with ranges from 4.3% to 83.3% reported [49]. The DESTINY-PanTumor02 trials examined the HER2-targeting ADC trastuzumab deruxtecan (T-Dxd) across a variety of cancer types, including bladder cancer, and found an objective response rate (ORR) of 56.3% in bladder cancer patients with immunohistochemical (IHC) staining of 3+ using gastric cancer scoring standards [50]. T-Dxd is an ADC using an antibody to the extracellular domain of the HER2 receptor, with a topoisomerase I inhibitor payload and a tetrapeptide-based cleavable linker.

Each ADC requires an understanding of its structure and special vigilance to unique side effects that result from its antibody target (on-target side effects in healthy tissues), linker (drug stability prior to and once reaching target tissues), payload (off-target side effects from systemic circulation of cytotoxic agents), and drug-to-antibody ratio (DAR). EV targets Nectin-4, which is expressed in skin cells and sweat glands, and demonstrates high rates of on-target skin toxicities, including low rates of life-threatening skin toxicities such as Stevens–Johnson Syndrome or Toxic Epidermal Necrolysis [51]. Additional on-target toxicities included ocular toxicity due to Nectin-4 expression in the corneal epithelium and conjunctiva. Furthermore, the payload MMAE is a microtubule-disrupting agent with known neurotoxicity, and both motor and sensory nerve toxicities are common, especially with prolonged exposure. Finally, some toxicities are unique to specific ADCs with poorly understood mechanisms, such as hyperglycemia in EV, a side effect that is often reversible with drug interruptions. SG has fewer on-target toxicities in healthy cells, though higher rates of hypersensitivity reactions to its TROP-2 antibody require premedication. The payload of SG, SN-38, is also the active metabolite of irinotecan and known to cause diarrhea and neutropenia. SN-38 is poorly metabolized by patients with specific UGT1A1 polymorphisms, requiring consideration of pharmacogenomic screening and careful monitoring with supportive care for diarrhea and neutropenia, with a recent guideline update recommending strong consideration of prophylactic growth factor administration with SG [52]. Most data from HER2-targeting ADCs comes from other cancer types, though the toxicities in available bladder cancer studies appear similar. For T-Dxd, these include low rates of on-target cardiotoxicity, off-target myelosuppression and gastrointestinal toxicity from the topoisomerase I inhibitor payload, and the serious potential side effect of pneumonitis occurring in around 10% of patients across tumor types, which can be fatal in a small subset of patients [53]. Important to note, as ADC/ICI combinations expand, is the overlapping toxicities of ADCs and ICIs, including skin, gastrointestinal, and pulmonary toxicities, which can be permanent and/or life-threatening and must be weighed against the compelling efficacy of these combinations [54].

#### 2.2.1. Nectin-4-Targeting ADCs and Combinations

The potential of ADCs in bladder cancer was first demonstrated by studies of EV and SG in patients with advanced urothelial carcinoma who progressed after prior chemotherapy and immune checkpoint inhibition [55,56]. Subsequently, these drugs moved into the perioperative space with the phase Ib/II EV-103 trial, which studied not only EV plus pembrolizumab combination therapies in cohorts with metastatic UC, but also EV monotherapy in perioperative cohorts H and L for patients with cisplatin-ineligible MIBC [34,35]. Though small cohorts, these arms showed pCR rates of 36.4% (8/22) and 34.0% (17/50), respectively. Cohort H involved only neoadjuvant EV, while cohort L studied a sandwich approach with both preoperative and postoperative EV cycles.

More recently, the phase III EV-303/KEYNOTE-905 trial investigating a sandwich EV plus pembrolizumab strategy (3 cycles of EV plus pembrolizumab combination therapy pre-operatively, and 6 cycles of EV and 14 cycles of pembrolizumab post-operatively) compared to cystectomy alone in patients with MIBC ineligible for or declining cisplatin [11]. This trial began as a perioperative sandwich pembrolizumab monotherapy trial, and the results from this arm have not yet been reported. However, following the success of EV plus pembrolizumab in the advanced setting, a third arm with EV plus pembrolizumab was added. Though this study initially enrolled only cisplatin-ineligible patients, the study was later amended to include patients who declined cisplatin, a population that made up 16.5% of patients in the EV plus pembrolizumab arm and 20.1% of the cystectomy arm. The results for pCR (57.1% vs. 8.6%, *p* < 0.001), DFS (HR 0.40, 95% CI 0.28–0.57, *p* < 0.001), and OS (HR 0.50, 95% CI 0.33–0.74, *p* < 0.001) all strongly favored the combination EV pembrolizumab arm leading to a rapid FDA approval of this combination in the perioperative space. In the EV/pembrolizumab arm, 71.3% of patients experienced a grade 3 or higher adverse event when compared to 45.9% in the control group. The most common adverse events in the EV/pembrolizumab arm were skin reactions (57.5%), pruritis (47.3%), alopecia (34.7%), and diarrhea (34.1%), with the most common grade 3 or higher adverse events including severe skin reactions, urinary tract infections, anemia, and diarrhea. Given the previously described unique toxicities of EV, toxicities were also examined specifically for peripheral neuropathy (36.5%), ocular disorders (17.4%), and hyperglycemia (9.6%).

Shortly after the publication of EV-303/KEYNOTE-905, results from the phase III EV-304/KEYNOTE-B15 of perioperative EV plus pembrolizumab (4 neoadjuvant cycles of both, 5 adjuvant cycles of EV and 13 adjuvant cycles of pembrolizumab) versus chemotherapy with gemcitabine and cisplatin (with adjuvant nivolumab allowed after February 2023) in cisplatin-eligible patients with MIBC were reported. This trial demonstrated an EFS benefit for the EV plus pembrolizumab arm with a median EFS not reached vs. 48.5 mo, and a HR of 0.53 (95% CI 0.41–0.70, *p* < 0.0001) [13]. The EV plus pembrolizumab arm also demonstrated improved OS, with a median OS not reached in either arm and a HR of 0.65 (95% CI 0.48–0.89, *p* = 0.0029), and pCR, with a rate of 55.8% vs. 32.5% (estimated difference of 23.4%, 95% CI 16.7–29.8%). Of the patients in the EV plus pembrolizumab arm, 75.7% experienced a grade 3 or higher toxicity, compared to 67.2% in the chemotherapy arm. The most common toxicities in the EV plus pembrolizumab arm were pruritis (46.2%), diarrhea (34.0%), and alopecia (31.5%), and 20.6% of patients experienced a rash. In the EV plus pembrolizumab arm, 85.4% of patients completed all four neoadjuvant cycles with at least one of the two study drugs, 88.2% of patients in the EV plus pembrolizumab arm underwent cystectomy, and of the 65% of patients who started the adjuvant phase, 79.4% completed either all 5 planned EV cycles or all 13 planned pembrolizumab cycles. Though no direct comparison to other ICI-containing perioperative regimens can be made, this EV304/KEYNOTE-B15 regimen is expected to become a standard of care for cisplatin-eligible MIBC patients electing for cystectomy.

Several ongoing trials may further elucidate the roles of EV combinations in MIBC. The phase III VOLGA trial includes three arms, one for radical cystectomy alone, one for EV plus durvalumab, and one for EV plus durvalumab plus tremelimumab (CTLA4 antibody) [36]. Interestingly, EV is only given pre-operatively, which may help us to understand the contribution of the postoperative ADC treatments in the EV-303/KEYNOTE-905 and EV-304/KEYNOTE-B15 studies, and how to balance the efficacy of these agents with known toxicities from prolonged exposure.

#### 2.2.2. TROP2- and HER2-Targeting ADCs and Combinations

Additionally, TROP2- and HER2-targeting ADCs remain of interest. In the locally advanced (1a)/metastatic (m) UC setting, the TROP2-targeting ADC, SG, was evaluated in two key trials. TROPHY-U-01 was a phase II, open-label study examining SG in the post-chemotherapy and post-immune checkpoint inhibitor setting for la/mUC, and reported an ORR of 28% (95 CI 20.2–37.6%) with mPFS of 5.4 months and mOS of 10.9 months [56], leading to initial approval of SG in the refractory setting. However, the manufacturer withdrew this SG indication after the subsequent phase III, randomized, TROPiCs-04 compared SG to physician’s choice chemotherapy (paclitaxel, docetaxel, or vinflunine) and failed to demonstrate improvement in its primary outcome of overall survival (mOS 10.3 vs. 9.0 mo, HR 0.86, 95% CI 0.73–1.02, *p* = 0.087) [57]. Of note, there were low rates of primary prophylactic granulocyte colony-stimulating factor (G-CSF, 21% in SG arm) that may have influenced outcomes with SG. In the perioperative setting, the phase II SURE-01 trial investigated preoperative SG for four cycles in cisplatin-ineligible or refusing patients. The trial showed a pCR rate of 36.4% and a 12-month EFS rate of 78.8% with 27.3% grade 3 or higher adverse events and one grade 5 (death) event [14]. SURE-02 is a phase II trial combining SG with pembrolizumab in a sandwich approach with 4 cycles of SG plus pembrolizumab followed by radical cystectomy followed by 13 additional cycles of pembrolizumab [32,33]. Amendments were made to the initial protocol to reduce the dose of SG (from 10 mg/kg to 7.5 mg/kg with each administration), include primary G-CSF prophylaxis, and exclude patients at high risk of neutropenia based on published toxicity reports from SG monotherapy and combination studies. Initial outcomes reported a clinical CR (cCR) rate of 39% (95% CI 25–54%), a 12-month EFS rate of 71.0% (95% CI 57–89%), and a 12-month bladder-intact EFS of 38% (95% CI 25–59%) with 16% grade 3 or higher adverse events. The authors also noted an increased response to SG/pembrolizumab in tumors with a luminal molecular signature.

HER2-targeting ADCs are gaining traction with improved outcomes relative to cisplatin-based chemotherapy in the 1a/mUC setting. The phase III, RC48-C016 study combined disitamab vedotin (DV, HER2-targeting ADC with a protease-cleavable linker and MMAE payload) with toripalimab (PD-1 inhibitor) compared with chemotherapy (gemcitabine plus cisplatin or carboplatin) and showed PFS (mPFS 13.1 vs. 6.5 mo, HR 0.36, 95% CI 0.28–046, *p* < 0.001) and OS (mOS 31.5 vs. 16.9 mo, HR 0.54, 95% CI 0.41–0.73, *p* < 0.001) benefits for patients with HER2+ mUC [58]. This benefit was consistent across both the HER2 IHC 2+/3+ subgroup and the HER2 IHC 1+ subgroup. Grade 3 or higher adverse events occurred in 55.1% of the DV/toripalimab arm and 86.9% of the chemotherapy arm, with important grade 3 or higher DV/toripalimab toxicities including increased gamma-glutamyltransferase (7.4%), hypokalemia (5.8%), decreased neutrophil count (5.8%), and nerve-related adverse events including hypoesthesia (38.7% any grade and 5.3% grade 3+) and peripheral neuropathy (21.8% any grade and 4.1% grade 3+). In the perioperative space, DV has been studied in multiple combinations, including with toripalimab in the phase II RC48-C017 study, where six cycles of DV plus toripalimab showed a pCR rate of 63.6% with 27.7% grade 3 or higher adverse events (including 17% adverse events requiring discontinuation) [15]. DV has also been combined with cadonilimab (PD-1/CTLA-4 bispecific antibody) in the phase II NCT06074484 trial, which showed a pCR rate of 64.7% and a grade 3 or higher toxicity rate of 15.4%, including one patient death from a cerebrovascular accident [16]. DV is not currently approved in the United States, but there are ongoing global trials for further registration attempts, such as a phase II trial investigating perioperative DV plus ivonescimab (PD-1/VEGF bispecific antibody) [38]. In addition, there is an ongoing global, phase III, registration trial of DV and pembrolizumab for HER2+ mUC (NCT05911295) [59]. These trials, along with the tumor-agnostic approval of trastuzumab deruxtecan, highlight the promise of HER2-targeting ADCs for future treatment of HER2-selected urothelial cancers. Finally, the aforementioned Interpath-005 trial includes an ADC cohort examining adjuvant pembrolizumab plus EV with or without a personalized mRNA vaccine (intismeran autogene or V940) [30].

## 3. Discussion

Perioperative immunotherapy and ADC approaches are revolutionizing the care of patients with bladder cancer. Here, we review the historical trials leading up to the current standard of care perioperative treatment approaches, including the NIAGARA regimen of perioperative durvalumab combined with cisplatin-based chemotherapy, the EV-303/ KEYNOTE-905 regimen of perioperative EV plus pembrolizumab in cisplatin-ineligible patients, the EV-304/KEYNOTE-B15 regimen of perioperative EV plus pembrolizumab in cisplatin-eligible patients, and adjuvant nivolumab or pembrolizumab with or without prior neoadjuvant chemotherapy. Given the robust improvements in clinical outcomes using ICI/ADC combinations, EV plus pembrolizumab is a new standard of care option in the perioperative setting, and ICI/ADC combinations are likely to continue shifting the MIBC therapeutic landscape.

Several limitations of existing ICI and ADC MIBC perioperative therapies are relevant to highlight. All large phase III trials evaluating perioperative ICI and ADC therapies are focused on urothelial-predominant histologies in bladder cancer, and robust data on the use of these therapies in primarily variant histology MIBC and upper-tract disease is lacking. Ongoing trials such as NCT05581589 examining neoadjuvant SG in variant histology bladder cancer and EA8192/NCT04628767 evaluating the addition of the ICI durvalumab in a sandwich perioperative approach to neoadjuvant chemotherapy in high-grade upper tract urothelial cancer will help elucidate the roles of ADCs and ICIs in these settings [60,61].

Furthermore, the necessity of the frequently used “sandwich” perioperative approach remains unclear, where predictive biomarkers of response/resistance, such as pCR and ctDNA, are needed to identify those most likely to benefit from additional therapy in the postoperative setting. Particularly, the clinical efficacy of these sandwich approaches must be balanced with the ongoing risk of toxicities associated with additional exposure (e.g., neuropathy from prolonged EV exposure and permanent endocrine toxicities from ICI). IMvigor011 highlights ctDNA positivity after cystectomy as a potential biomarker of benefit from adjuvant ICI therapy. The ongoing VOLGA study, which only included EV in the neoadjuvant setting, may grant some insight into the role of adjuvant ICI/ADC versus ICI. Ongoing knowledge of efficacy and toxicity should be incorporated into clinical practice and clinical trial designs.

Finally, in patients who achieve remarkable responses with preoperative systemic therapies, the necessity of radical cystectomy for all patients with MIBC is called into question. Particularly, the combination of more effective, ADC/ICI perioperative systemic therapies and improved surveillance technologies (such as standardization of pCR definitions, improved ctDNA minimal residual disease detection, and multiparametric MRI) highlights the immense opportunity for bladder-sparing approaches when appropriate criteria are met [62]. Thus far, several completed, bladder-sparing perioperative clinical trials highlight the feasibility of conducting these needed investigations. Worth noting are the RETAIN-1 trial utilizing precision oncology approaches (patients whose tumors harbored homologous recombination repair deficiency receiving platinum-based neoadjuvant chemotherapy) and the RETAIN-2 and HCRN GU16-257 trials evaluating perioperative chemoimmunotherapy combinations (combination atezolizumab and nivolumab with chemotherapy, respectively) [63,64,65]. Precision medicine insights from trials such as SURE-02 and CheckMate 274, ctDNA monitoring results from NIAGARA and IMvigor011, and ongoing trials of trimodal therapy involving ICI/ADC highlight further opportunities to refine appropriate candidates for bladder preservation.

## 4. Conclusions

ICIs and ADCs have revolutionized the treatment of patients with MIBC undergoing radical cystectomy, offering unprecedented disease-free survival and overall survival outcomes. Current FDA-approved standards of care for perioperative MIBC therapies include perioperative durvalumab combined with neoadjuvant cisplatin-based chemotherapy, perioperative sandwich EV plus pembrolizumab in patients with cisplatin-ineligible MIBC, and multiple adjuvant ICI therapy options for patients with high-risk features with or without prior neoadjuvant chemotherapy. Based on recently presented data, perioperative sandwich EV plus pembrolizumab in cisplatin-eligible patients with MIBC is positioned to become another standard of care option for this disease. Indications for ICI and ADC therapies in MIBC are expected to continue to expand in the coming years, with many ongoing trials in this space, and ongoing investigation of biomarker-selected precision oncology approaches will continue to shape the therapeutic landscape of perioperative MIBC.

## Figures and Tables

**Table 1:** Select MIBC trials of perioperative immunotherapy with or without chemotherapy.

***Reported Trials***								
**Trial**	**Design**	**Neoadjuvant Therapy**	**Adjuvant** **Therapy**	**Key Inclusion** **Criteria**	**pCR Rate**	**mDFS/mEFS**	**mOS**	**Grade 3+** **Treatment-Related Toxicity**
NIAGARA [17]	Phase III N = 533 vs. 530	Gemcitabine + cisplatin +/− durvalumab	Durvalumab vs. no adj. treatment	cT2-T4a, N0-1 CrCl ≥ 40 mL/min	33.8% vs. 25.8%, RR 1.30 (95% CI 1.09–1.56, *p* = 0.004)	NR vs. 46.1 mo HR 0.68 (95% CI 0.56–0.82, *p* < 0.001)	NR vs. NR, HR 0.75 (95% CI 0.59–0.93, *p* = 0.01)	40.6% vs. 40.9%
CheckMate 274 [18]	Phase III N = 353 vs. 356	Cisplatin-based chemotherapy allowed (43.3% vs. 43.5%)	Nivolumab vs. no adj. treatment	ypT2-T4a or ypN+; pT3-T4a or pN+	N/A	21.9 vs. 11.0 mo, HR 0.74 (95% CI 0.61–0.90)	75.0 vs. 50.1 mo, HR 0.83 (95% CI 0.67–1.02)	18.2% vs. 7.2%
AMBASSADOR [19]	Phase III N = 354 vs. 348	Cisplatin-based chemotherapy allowed (64.7% vs. 62.6%)	Pembrolizumab vs. no adj. treatment	ypT2-T4a or ypN+; pT3-T4a or pN+; any microscopic positive margins	N/A	29.6 vs. 14.2 mo, HR 0.73 (95% CI 0.59–0.90, *p* = 0.003)	NR-NR, HR 0.98 (95% CI 0.76–1.26)	24.3% (intervention arm)
IMvigor011 [20]	Phase III N = 761 enrolled; N = 250 ctDNA+ (N = 167 vs. 83)	Neoadjuvant chemotherapy allowed (47.9% vs. 39.8%)	Atezolizumab vs. placebo	pT2-T4 or pN+, ypT2-T4a or ypN+; selected for ctDNA+	N/A	9.9 vs. 4.8 mo, HR 0.64 (95% CI 0.47–0.87; *p* = 0.0047)	32.8 vs. 21.1 mo, HR 0.59 (95% CI 0.39, 0.90; *p* = 0.0131)	7.3% vs. 3.6%
IMvigor010 [21]	Phase III N = 409 vs. 403	Cisplatin-based chemotherapy allowed (48% vs. 47%)	Atezolizumab vs. no adj. treatment	ypT2-T4a or ypN+; pT3-T4a or pN+	N/A	19.4 vs. 16.6 mo, HR 0.89 (95% CI 0.74–1.08, *p* = 0.24)	NR vs. NR, HR 0.85 (90% CI 0.66–1.09 )	16% (intervention arm)
SunRISe-4 [22]	Phase II N = 159	TAR-200 + cetrelimab vs. cetrelimab	N/A	cT2-T4aN0M0	42% vs. 23%	Not mature	N/A	11% vs. 5%
SAKK 06/19 (NCT04630730) [23]	Phase II, N = 47	Intravesical BCG × 3 + atezolizumab × 4 + GC × 4	Atezolizumab × 13	pT2 or cT2-4aN0-1	42.9% (9/21 patients evaluable to date)	Not yet available	Not yet available	9% from BCG, 17% IRAE, 38% chemo
AURA Oncodistinct-004 [24]	Phase II N = 79 Phase II N = 58	Avelumab + ddMVAC vs. GC Avelumab +/− PG	N/A N/A	cT2-T4a, cN0-N1, Cisplatin- eligible cT2-T4a, cN0-N1, Cisplatin- ineligible	58% vs. 53% 14% vs. 42%	NR vs. NR NR vs. NR	NR vs. NR NR vs. NR	56% across arms (3% IRAEs) 12.5% across arms
SAKK 06/17 [25]	Phase II N = 57	4 cycles GC + durvalumab	10 cycles durvalumab	cT2-T4a, cN0-1, cisplatin- eligible	33%	EFS at 2 years 75.7%	OS at 2 years 85%	67% neoadj., 15% adj.
PURE-01 [26]	Phase II N = 155	Pembrolizumab (+ subsequent therapy in 14)	N/A	cT2-T4N0	36.8%	NR	NR	4.5%
ABACUS [27]	Phase II, N = 95	Atezolizumab	N/A	cT2-T4N0, Cisplatin- ineligible	31%	NR	NR	N/A (11 overall grade 3+ toxicities)
***Ongoing Trials***								
**Trial**	**Design**	**Neoadjuvant Therapy**	**Adjuvant** **Therapy**	**Key Inclusion** **Criteria**	**ClinicalTrials.gov ID**	**Estimated** **Completion**		
KEYNOTE-866 [28]	Phase III, N = 907	Neoadjuvant GC +/− pembrolizumab	Pembrolizumab vs. no adj. treatment	cT2-T4aN0 or cT1-T4aN1	NCT03924856	September 2026		
MODERN [29]	Phase II/III, N = 992	Neoadjuvant chemotherapy allowed	ctDNA+: nivolumab +/− relatlimab ctDNA−: nivolumab vs. ctDNA monitoring	pT3-T4 or pN+, ypT2-T4a or ypN+	NCT05987241	September 2030		
INTerpath-005 (Adjuvant Cohort) [30]	Phase I/II, N = 230	Neoadjuvant chemotherapy allowed	Pembrolizumab +/− V940	MIBC (>pT2)	NCT06305767	October 2031		

Adj.—adjuvant; CI—confidence interval; CrCl—creatinine clearance; ctDNA+—circulating tumor DNA positive; ddMVAC—dose-dense methotrexate–vinblastine–doxorubicin–cisplatin; GC—gemcitabine–cisplatin; HR—hazard ratio; IRAE—immune-related adverse event; mDFS—median disease-free survival; mEFS—median event-free survival; mo—month; mOS—median overall survival; N/A—not available; NR—not reached; Neoadj.—neoadjuvant; pCR—pathologic complete response; PG—paclitaxel–gemcitabine; RR—risk ratio.

**Table 2:** Select MIBC Clinical Trials of Perioperative ADC with or without ICI.

***Reported Trials***								
**Trial**	**Design**	**Neoadjuvant Therapy**	**Adjuvant** **Therapy**	**Key Inclusion** **Criteria**	**pCR Rate**	**mDFS/mEFS**	**mOS**	**Grade 3+** **Treatment-Related Toxicity**
EV-304/KEYNOTE-B15 [13,31]	Phase III, N = 405 vs. 403	EV+ Pembrolizumab × 4 vs. GC	EV × 5 + Pembrolizumab × 13 vs. no adj. treatment	cT2-T4aN0 or cT1-T4aN1 cisplatin-eligible	55.8% vs. 32.5%, *p* < 0.0001	NR vs. 48.5 mo, HR 0.53 (95% CI 0.41–0.70, *p* < 0.001)	NR vs. NR, HR 0.65 (95% CI 0.48–0.89, *p* = 0.0029)	75.7% * vs. 67.2% *
EV-303/KEYNOTE-905 [11,12]	Phase III, N = 170 vs. 174	3 cycles EV+ pembrolizumab vs. no neoadj. treatment	6 cycles EV + 14 cycles pembrolizumab vs. no adj. treatment	cT2-4aN0-1M0, cisplatin-ineligible or declined (16.5 vs. 20.1%)	57.1% vs. 8.6%, *p* < 0.001	NR vs. 15.7 mo, HR 0.40 (95% CI 0.28–0.57, *p* < 0.001)	NR vs. 41.7 mo, HR 0.50 (95% CI 0.33–0.74, *p* < 0.001)	71.3% * (45.5% treatment-related) vs. 45.9% *
SURE-01 [14]	Phase II, N = 37	4 cycles SG	No adj. treatment	cT2-T4N0M0 cisplatin-ineligible or declined	36.4% (95% CI: 20.4–54.9%)	12-month EFS 78.8% (95% CI 66–94%)	N/A	27.3%
SURE-02 [32,33]	Phase II, N= 48	SG + pembrolizumab × 4	Pembrolizumab × 13	cT2-T3bN0M0	cCR Rate 39% (95% CI 25–54%)	12-month EFS 71% (95% CI 57–89%)	12-month bladder-intact EFS 38% (25–59%)	16.3%
RC48-C017 [15]	Phase II, N = 47	6 cycles DV + toripalimab	20 cycles toripalimab	cT2-4aN0-1M0, HER2 IHC ≥ 1+	63.6% (95% CI: 45.1–79.6%)	1-year EFS 92.5% (95% CI: 72.8–98.1%).	1 year OS 95.5% (95% CI: 83.3–98.9%).	21.3%
NCT06074484 [16]	Phase II, N = 43	4 cycles DV + cadonilimab	6 cycles DV + 14 cycles cadonilimab	cT2-4aN0-1M0, HER2 IHC ≥ 1+	64.7% (95% CI 47.9–78.6%)	N/A	N/A	18.6%
EV-103 Cohort H [34]	Phase Ib/II, N = 22	3 cycles EV	No adj. treatment	cT2-T4aN0 cisplatin-ineligible	36.4% (*n* = 8/22, 95% CI 17.2–59.4%)	40.1 mo (95% CI 14.5-NE)	NR (95% CI 33.4 mo-NE)	13.6% discontinuation rate
EV-103 Cohort L [35]	Phase Ib/II, N = 51	3 cycles EV	6 cycles EV	cT2-T4aN0 or cT1-T4aN1, cisplatin-ineligible	34.0% (n = 17/50)	N/A	N/A	39.2%
***Ongoing Trials***								
**Trial**	**Design**	**Neoadjuvant Therapy**	**Adjuvant** **Therapy**	**Key Inclusion** **Criteria**	**Enrollment Goal**	**ClinicalTrials.gov ID**	**Estimated** **Completion**	
VOLGA [36]	Phase III, N = 712	EV + durvalumab × 3 +/− tremelimumab × 2 vs. no neoadj. treatment	Durvalumab × 9 +/− tremelimumab × 1 vs. vs. no adj. treatment	cT2-T4aN0/1 or T1N1	712	NCT04960709	September 2028	
EV-ECLIPSE [37]	Phase II, N = 23	EV + pembrolizumab × 6	Pembrolizumab × 11	cT2-T4, N1-N3 or cT1, N2-N3	23	NCT05239624	June 2026	
NCT06957561 [38]	Phase II, N = 30	DV × 6 + Ivonescimab × 4	DV+ Ivonescimab × 9	cT2-T4aN0-1 M0 Cisplatin-ineligible or declining	30	NCT06957561	May 2029	
INTerpath-005 (Perioperative Cohort) [30]	Phase I/II, N = 230	EV + pembrolizumab × 4 +/− V940 × 1−4	EV x 5+ pembrolizumab x13 +/− V940 × 5−8	MIBC (>pT2)	230	NCT06305767	October 2031	

Adj.—adjuvant; cCR—clinical complete response; CI—confidence interval; DV—disitamab vedotin; EV—enfortumab vedotin; GC—gemcitabine plus cisplatin; HR—hazard ratio; mDFS—median disease-free survival; mEFS—median event-free survival; mo—month; mOS—median overall survival; N/A—not available; NE—not estimatable; Neoadj.—neoadjuvant; NR—not reached; pCR—pathologic complete response; SG—sacituzumab govitecan. * Treatment-emergent adverse event rate reported as treatment-related rate not available.

**Table 3:** Biases and limitations of phase III ICI and ADC perioperative MIBC clinical trials.

Trial	Design	Neoadjuvant Therapy	Adjuvant Therapy	Key Inclusion Criteria	Biases/Limitations
NIAGARA [17]	Phase III N = 533 vs. 530	Gemcitabine + cisplatin +/− durvalumab	Durvalumab vs. no adj. treatment	cT2-T4a, N0-1 CrCl ≥ 40 mL/min	Open-label, lack of adjuvant ICI in control arm
CheckMate 274 [18]	Phase III N = 353 vs. 356	Cisplatin-based chemotherapy allowed (43.3% vs. 43.5%)	Nivolumab vs. no adj. treatment	ypT2-T4a or ypN+; pT3-T4a or pN+	Lack of statistically significant OS benefit in 5-year analysis
AMBASSADOR [19]	Phase III N = 354 vs. 348	Cisplatin-based chemotherapy allowed (64.7% vs. 62.6%)	Pembrolizumab vs. no adj. treatment	ypT2-T4a or ypN+; pT3-T4a or pN+; any microscopic positive margins	Open-label, lack of OS benefit
IMvigor011 [20]	Phase III N = 761 enrolled; N = 250 ctDNA+ (N = 167 vs. 83)	Neoadjuvant chemotherapy allowed (47.9% vs. 39.8%)	Atezolizumab vs. placebo	pT2-T4 or pN+, ypT2-T4a or ypN+; selected for ctDNA+	Only applies to ctDNA-positive population
IMvigor010 [21]	Phase III N = 409 vs. 403	Cisplatin-based chemotherapy allowed (48% vs. 47%)	Atezolizumab vs. no adj. treatment	ypT2-T4a or ypN+; pT3-T4a or pN+	Negative trial for primary outcome
EV-304/ KEYNOTE-B15 [13,31]	Phase III, N = 405 vs. 403	EV+ Pembrolizumab × 4 vs. GC	EV × 5 + Pembrolizumab x13 vs. no adj. treatment	cT2-T4aN0 or cT1-T4aN1 cisplatin-eligible	No direct comparison to other ICI-containing perioperative regimens
EV-303/KEYNOTE-905 [11,12]	Phase III, N = 170 vs. 174	3 cycles EV+ pembrolizumab vs. no neoadj. treatment	6 cycles EV +14 cycles pembrolizumab vs. no adj. treatment	cT2-4aN0-1M0, cisplatin-ineligible or declined (16.5 vs. 20.1%)	Complex, shifting multi-arm design, e.g., cisplatin-ineligible, later expanded to cisplatin-declining

Adj.—adjuvant; EV—enfortumab vedotin; GC—gemcitabine plus cisplatin; Neoadj.—neoadjuvant.

## Data Availability

No new data were created or analyzed in this study.

## Abbreviations

The following abbreviations are used in this manuscript:

ADC	antibody–drug conjugate
cCR	clinical complete response
CI	confidence interval
ctDNA	circulating tumor DNA
CrCl	creatinine clearance
ddMVAC	dose-dense methotrexate–vinblastine–doxorubicin–cisplatin
DFS	disease-free survival
DV	disitimab vedotin
EFS	event-free survival
EV	enfortumab vedotin
HER2	epidermal growth factor receptor 2
HR	hazard ratio
ICI	immune checkpoint inhibitor
IRAE	immune-related adverse event
la	locally advanced
MIBC	muscle-invasive bladder cancer
mDFS	median disease-free survival
mEFS	median event-free survival
mOS	median overall survival
N/A	not available
NE	not estimatable
NR	not reached
OS	overall survival
pCR	pathologic complete response
PG	paclitaxel–gemcitabine
RR	risk ratio
SG	sacituzumab govitecan
Trop-2	tumor-associated calcium signal transducer 2
UC	urothelial carcinoma
